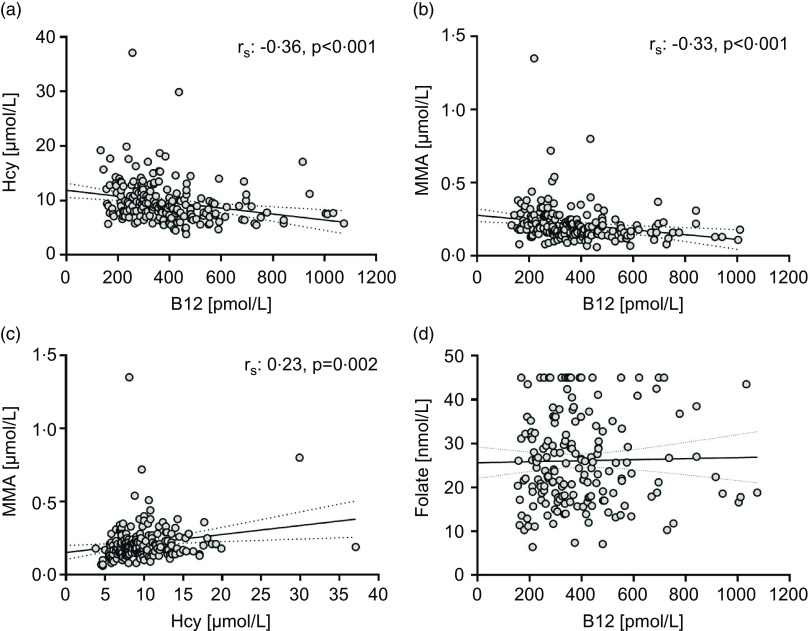# A response to ‘Highlights of the importance of vitamin B_12_ for neurological and cognitive function: from pregnancy to childhood’

**DOI:** 10.1017/S0007114522004020

**Published:** 2023-09-28

**Authors:** Sigrun Henjum

**Affiliations:** Department of Nursing and Health Promotion, Faculty of Health Sciences, Oslo Metropolitan University, Postboks 4, St. Olavs plass 0130 Oslo, Norway

Dear Editor

Here are my responses to the Letter from Dr P. Zanella entitled ‘Highlights of the importance of vitamin B_12_ for neurological and cognitive function: from pregnancy to childhood’^(1)^.

1. Dr Zanella: First, it is reported in the Methodology that the participants were between 18 and 60 years old, but in the Discussion, they say that in their sample, only three participants were over 60 years old.
  Response: In the Method section, it was reported that the study sample consisted of 205 participants, 115 vegans and 90 vegetarians from the Oslo area (57 men and 148 women, age range 18–60 years). The age range is reported to present the min and max age. The mean age of the participants is also reported in Table 1.
  Due to few previous studies measuring biomarkers for B_12_ status in adult vegans and vegetarians, we compared our findings against a study with elderly people^(2)^.
  However, we point out that our study is not directly comparable to this study, as only three participants were above 60 years. The study is also compared against a study in adults having a vegetarian diet^(3)^.
2. Dr Zanella: As the body storage of vitamin B_12_ is quite long, a greater restriction would probably result in different results for the study.
  Response: The mean diet duration in this study was 4·7 years, as reported in Table 1. Thus, we believe that the study results would not have been much different with 1 year as an inclusion criterion, as most of the participants had restricted their diets for several years.
3. Dr Zanella: Third, the study considers serum vitamin B_12_ adequate above 221 pmol/l, but perhaps an analysis with a more rigorous cut-off point would bring interesting results given its importance for health.
  Response: We used classical cut-offs for evaluation of B_12_ status: severely deficient (≤ 148 pmol/l), marginally deficient (149 to 221 pmol/l), deficient (< 221 pmol/l) and adequate (> 221 pmol/l). A single biomarker-like serum B_12_ is not a definitive indicator of B_12_ status or deficiency; if low, it suggests other markers should be used as well. Methylmalonic acids are the most sensitive, followed by holoTC, then B_12_ and then homocysteine. cB12, as used in our study, includes several of these and is therefore specific and sensitive to detect true deficiency. It is quite common for people to be diagnosed as B_12_ deficient and yet be asymptomatic, not least because symptoms are not usually detected until serum B_12_ is very low, i.e. probably < 125 pmol/l. We reported the number of persons with B_12_ levels below 148 pmol/l, which was only two participants (one vegan and one vegetarian).
4. Dr Zanella: As a fourth question, the authors mention Fig. 1 in the results, but none is presented in the study.
  Response: Thank you for this comment. Fig. 1 is now included.

**Fig. 1.**